# Search for New Participants in the Pathogenesis of High-Grade Serous Ovarian Cancer with the Potential to Be Used as Diagnostic Molecules

**DOI:** 10.3390/life12122017

**Published:** 2022-12-03

**Authors:** Angelika V. Timofeeva, Aleksandra V. Asaturova, Maya V. Sannikova, Grigory N. Khabas, Vitaliy V. Chagovets, Ivan S. Fedorov, Vladimir E. Frankevich, Gennady T. Sukhikh

**Affiliations:** 1 National Medical Research Center for Obstetrics, Gynecology and Perinatology Named after Academician V.I. Kulakov Ministry of Healthcare of The Russian Federation, Ac. Oparina 4, 117997 Moscow, Russia; 2 Laboratory of Translational Medicine, Siberian State Medical University, 634050 Tomsk, Russia

**Keywords:** miRNA, new-generation sequencing (NGS), quantitative RT-PCR, high-grade serous ovarian carcinoma (HGSOC), cytoreduction, response to chemotherapy, progesterone receptor

## Abstract

Recent studies have attempted to develop molecular signatures of epithelial ovarian cancer (EOC) based on the quantitation of protein-coding and non-coding RNAs to predict disease prognosis. Due to the heterogeneity of EOC, none of the developed prognostic signatures were directly applied in clinical practice. Our work focuses on high-grade serous ovarian carcinoma (HGSOC) due to the highest mortality rate relative to other types of EOC. Using deep sequencing of small non-coding RNAs in combination with quantitative real-time PCR, we confirm the dualistic classification of epithelial ovarian cancers based on the miRNA signature of HGSOC (type 2), which differs from benign cystadenoma and borderline cystadenoma—precursors of low-grade serous ovarian carcinoma (type 1)—and identified two subtypes of HGSOC, which significantly differ in the level of expression of the progesterone receptor in the tumor tissue, the secretion of miR-16-5p, miR-17-5p, miR-93-5p, miR-20a-5p, the level of serum CA125, tumor size, surgical outcome (optimal or suboptimal cytoreduction), and response to chemotherapy. It was found that the combined determination of the level of miR-16-5p, miR-17-5p, miR-20a-5p, and miR-93-5p circulating in blood plasma of patients with primary HGSOC tumors makes it possible to predict optimal cytoreduction with 80.1% sensitivity and 70% specificity (*p* = 0.022, TPR = 0.8, FPR = 0.3), as well as complete response to adjuvant chemotherapy with 77.8% sensitivity and 90.9% specificity (*p* = 0.001, TPR = 0.78, FPR = 0.09). After the additional verification of the obtained data in a larger HGSOC patient cohort, the combined quantification of these four miRNAs is proposed to be used as a criterion for selecting patients either for primary cytoreduction or neoadjuvant chemotherapy followed by interval cytoreduction.

## 1. Introduction

In recent years, ovarian cancer ranks seventh in the order of overall cancer incidence, fifth among the causes of death from all malignant tumors in women [1], and is the leading cause of gynecological cancers according to the International Agency for Research on Cancer (IARC). Epithelial ovarian cancer (EOC) accounts for 60% of all cases of diagnosed ovarian cancer, is a heterogeneous group of carcinomas and consists of several histological subgroups, among which the most common are high-grade serous carcinoma, low-grade serous carcinoma, endometrioid carcinoma, clear cell carcinoma and mucinous carcinoma, each characterized by individual molecular genetic characteristics [2,3]. Among them, the serous type accounts for 75–80% of epithelial malignant neoplasms, and most serous carcinomas are diagnosed at already advanced stages of the disease. The highest mortality (about 70%) is observed among patients with high-grade serous ovarian carcinoma (HGSOC).

There are no clinical diagnostic and molecular biological methods for the early detection of EOC. EOC is characterized by high tumor heterogeneity and genomic instability, changes in the methylation status of the promoters of protein-coding genes and non-coding regions of the genome, and changes in the expression level of both proteins and their regulators at the post-transcriptional level [4,5,6,7,8,9,10,11,12], which provide multicomponent and multilevel pathogenesis of EOC and complicates the search for marker molecules to characterize this disease. HGSOC is characterized by mutations in the *TP53* gene in 96% of cases with or without somatic mutations in the *NF1*, *BRCA1*, *BRCA2*, *RB1*, and *CDK12* genes, 113 significant DNA copy number aberrations and changes in the methylation status of 168 genes, associated with the differential expression of 253 genes. Point mutations are much less common for low-grade serous ovarian carcinoma (LGSOC), in which the most frequent mutations are in *BRAF* and *KRAS* genes. A more complete and detailed molecular biological study of EOC is needed to identify new target molecules, which will serve as a basis for the development of methods for early diagnosis and effective therapeutic treatment of this disease.

Currently used diagnostic markers (including CA125 and HE4) do not have sufficient sensitivity and specificity to detect EOC [13]. It is known that increased levels of CA125 in the blood serum can also be detected in patients with endometriosis, adenomyosis, uterine fibroids, benign cysts, or inflammatory diseases in the pelvis. For example, in a retrospective analysis of serum samples from 5500 women in Sweden, an increase in CA125 levels was detected in 175 women, of whom only six were diagnosed with ovarian cancer, while three women with normal CA125 levels were diagnosed with ovarian cancer [14].

The main regulators of genome stability and gene expression at the epigenetic and post-transcriptional levels are small non-coding RNAs, including microRNAs (miRNAs). The same miRNA can be involved in the regulation of hundreds of target genes, while each of the structural genes is a target for different miRNAs [15]. Theoretically, the expression of 60% of human genes is under miRNA control [16]. Most miRNAs have oncogenic or oncosuppressive activity and can regulate various biological processes, including cell metabolism, proliferation, apoptosis, and chemoresistance [17,18]. Since miRNA expression is tissue-specific, detectable in blood, and correlates with clinical manifestations of cancer, miRNA can be used as potential diagnostic and prognostic tumor markers [19,20,21,22,23,24].

Many research teams have worked on the creation of a molecular portrait of HGSOC, comparing miRNA expression profiles in ovarian carcinoma with normal ovarian tissue or cell lines derived from the surface epithelium of the ovaries [25,26,27,28]. However, according to modern concepts of the pathogenesis of serous ovarian cancer, based on morphological and molecular genetic studies, the most likely source of development of serous ovarian carcinomas is the epithelium of the fallopian tube fimbriae [29,30,31,32]. There are two main pathways for the pathogenesis of serous carcinomas. One of them is the dissemination of fallopian tube epithelial stem cells to the surface of the ovary (presumably at the site of ovulation) with the formation of cystic inclusions, which can increase in volume with the formation of benign serous cystadenoma (BSC) with subsequent transformation into a serous borderline tumor (SBT) and low-grade serous carcinoma (LGSOC). The formation of HGSOC presumably occurs according to a different mechanism and begins with the secretory cells’ outgrowth with the occurrence of a mutation in the TP53 (p53 signature) and the formation of a serous tubal intraepithelial lesion (STIL). With the acquisition of additional somatic mutations, serous tubal intraepithelial carcinoma (STIC) is formed, the cells of which disseminate to the surface of the ovary and form a tumor.

In order to test two hypotheses of the pathogenesis of serous ovarian tumors, miRNA expression profiles in the BSC, SBT and HGSOC samples were compared relative to the that in samples of histologically unchanged fallopian tube fimbriae by new-generation sequencing (NGS). The top miRNAs from the signature of HGSOC were evaluated for specificity and potential for use as diagnostic molecules while comparing their expression level in the peripheral blood plasma of patients with various ovarian tumors or endometriosis.

## 2. Materials and Methods

### 2.1. Patients

In total, 122 women aged between 25 and 77-years-old were enrolled in the study and comprised the following groups: apparently healthy, n = 26; endometriosis, n = 24; benign serous cystadenoma, n = 18; borderline serous cystadenoma, n = 21; low-grade serous ovary cancer, n = 10; high-grade serous ovary cancer, n = 23 (Section 3.1 and Section 3.3). Written informed consent was obtained from each patient and the study was approved by the ethics committee of the National Medical Research Center for Obstetrics, Gynecology, and Perinatology, named after Academician V.I. Kulakov of the Ministry of Healthcare of the Russian Federation.

### 2.2. RNA Isolation from Peripheral Blood Plasma

Venous blood samples from women were collected into S-MONOVETTE tubes containing EDTA KE (Sarstedt AG&Co., Ltd., Nümbrecht, Germany, cat. No. 04.1915.100), centrifuged for 20 min at 300× *g* (4 °C), followed by plasma collection and re-centrifugation for 10 min at 16,000× *g*. RNA was extracted from 200 µL of blood plasma using an miRNeasy Serum/Plasma Kit (Qiagen, Germany, cat. No. 217184).

### 2.3. RNA Isolation from Fimbriae and Ovary Tumor Tissues

Fimbriae and ovary tumor tissues were collected for study during surgery and immediately frozen in liquid nitrogen for subsequent storage at –80 °C. Total RNA was extracted from 5–40 mg of tissue using the miRNeasy Micro Kit (Qiagen, Hilden, Germany, catalog No. 217084), followed by the RNeasy MinElute Cleanup Kit (Qiagen, Germany, catalog No. 74204). The RNA concentration was measured using the Qubit fluorometer 3.0 (Life Technologies, Petaling Jaya, Malaysia, cat.Q33216). The sample quality of the total RNA was examined on the Agilent Bioanalyzer 2100 (Agilent, Waldbronn, Germany, cat. No G2939A) using the RNA 6000 Nano Kit (Agilent Technologies, Santa Clara, CA, USA, cat. No. 5067-1511). Total RNA samples with an RNA integrity number (RIN) of at least 8 were used for further study.

### 2.4. miRNA Deep Sequencing

cDNA libraries were synthesized using 500 ng of total RNA from the fimbriae and ovary tumor tissues using the NEBNext^®^ Multiplex Small RNA Library Prep Set for Illumina^®^ (Set11 and Set2, New England Biolab^®^, Frankfurt am Main, Germany, cat. No. E7300S, E7580S), amplified for 14 and 18 PCR cycles, respectively, and sequenced on the NextSeq 500 platform (Illumina, San Diego, AC, USA, cat. No. SY-415-1001). The adapters were removed with Cutadapt. All trimmed reads shorter than 16 bp and longer than 30 bp were filtered, and only reads with a mean quality higher than 15 were retained. The remaining reads were mapped to the GRCh38.p15 human genome and miRBase v21 with the bowtie aligner [33]. Aligned reads were counted with the featureCount tool from the Subread package [34] and with the fracOverlap 0.9 option, so the whole read was forced to have a 90% intersection with sncRNA features. Differential expression analysis of the sncRNA count data was performed with the DESeq2 package [35].

### 2.5. Reverse Transcription and Quantitative Real-Time PCR

Seven microliters from 14 µL of total RNA column eluate (miRNeasy Serum/Plasma Kit, Qiagen, Germany, cat. No. 217184) extracted from 200 µL of blood plasma, or 250 ng of total RNA from the fimbriae or ovary tumor tissue, were converted into cDNA in a reaction mixture (20 µL) containing 1× Hispec buffer, 1× Nucleics mix, and miScript RT, in accordance with the miScript^®^ II RT Kit protocol (Qiagen, Germany, cat. No. 218161); then, the sample volume was adjusted with deionized water to 200 µL. The synthesized cDNA (2 µL) was used as a template for real-time PCR using a forward primer specific for the studied RNA (Table 1) and the miScript SYBR Green PCR Kit (Qiagen, Germany, cat. No. 218075). The following PCR conditions were used: (1) 15 min at 95 °C and (2) 40 cycles at 94 °C for 15 s, an optimized annealing temperature (52–62 °C) for 30 s and 70 °C at 30 s in a StepOnePlusTM thermocycler (Applied Biosystems, Waltham, MA, USA, cat. No. 4376600). The relative expression of miRNA in the blood plasma sample was determined by the ∆Ct method using miR-30d-5p as the reference RNA. The relative expression of miRNA in the tissue sample was determined by the ∆Ct method using SNORD68 as the reference RNA.

### 2.6. Immunohistochemistry

Tissue samples were fixed using 10% buffered formalin solution. Four to five-micrometer sections of formalin-fixed paraffin-embedded specimens were cut and immunohistochemical staining was performed with an automated immunostainer Ventana Benchmark Ultra and the prescribed Ventana protocol for progesterone receptor (PgR) staining. Monoclonal antibodies against PgR (clone 1E2) manufactured by Ventana were used, recognized A and B isoforms of PgR. Immunohistochemical results were evaluated in a semi-quantitative manner and scored according to intensity and the percentage of positively stained nuclei with an Allred scale. The Allred scale is a clinical instrument based on the percentage of cells that are stained by immunohistochemistry for steroid receptors (on a scale of 0 to 5) and the intensity of that staining (on a scale of 0 to 3, for a possible total score of 8 [36]. Appropriate controls were included.

### 2.7. Statistical Analysis of the Obtained Data

For statistical processing, scripts written in R language [34] and RStudio [37] were used. The correspondence of the analyzed parameters to the normal distribution law was assessed by the Shapiro–Wilk test. When the distribution of data was different from normal, the Mann–Whitney test for paired comparison was used, and data were described as the median (Me) and Q1 and Q3 quartiles in the format Me (Q1; Q3). Since both quantitative and qualitative characteristics were analyzed, a correlation analysis was performed using Spearman’s nonparametric correlation test. The 95% confidence interval for the correlation coefficient was determined using the Fisher transformation. The value of the threshold significance level (*p*) was taken as equal to 0.05. If the *p* value was less than 0.001, then *p* was indicated in the format *p* < 0.001.

## 3. Results

### 3.1. miRNA Signatures of the BSC, SBT and HGSOC According to NGS Data

In order to test two hypotheses of the pathogenesis of serous ovarian tumors, tissue samples from patient cohort 1 (Table 2) were used to compare miRNA expression profiles in the BSC, SBT and HGSOC samples with that in samples of histologically unchanged fallopian tube fimbriae by NGS.

**Figure 1:**
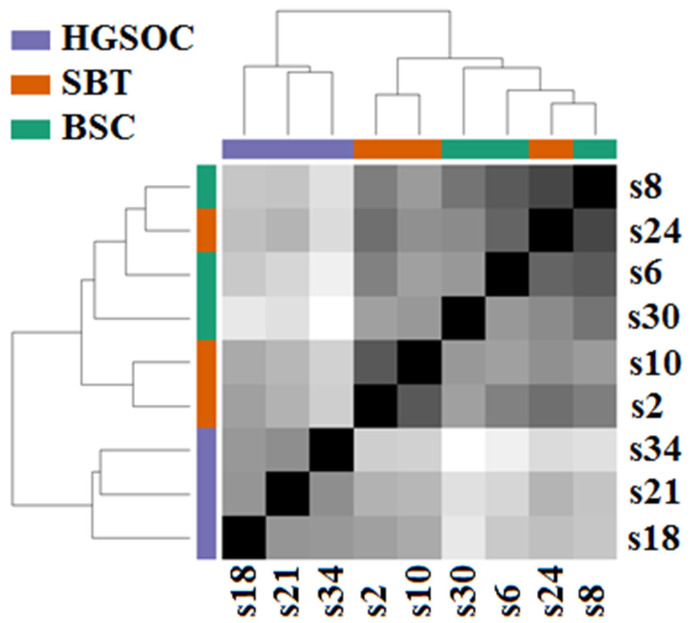
Hierarchical clustering of NGS data on read counts of miRNA from BSC (s6, s8, s30), SBT (s2, s10, s24,) and HGSOC (s18, s21, s34) samples.

The obtained miRNA expression profiles were analyzed by the hierarchical clustering method, and it was found that samples of HGSOC (s18, s21, s34) form a separate cluster, which differs markedly from the second cluster formed by SBT samples (s2, s10, s24) and BSC samples (s6, s8 s30), as demonstrated in Figure 1.

**Figure 2:**
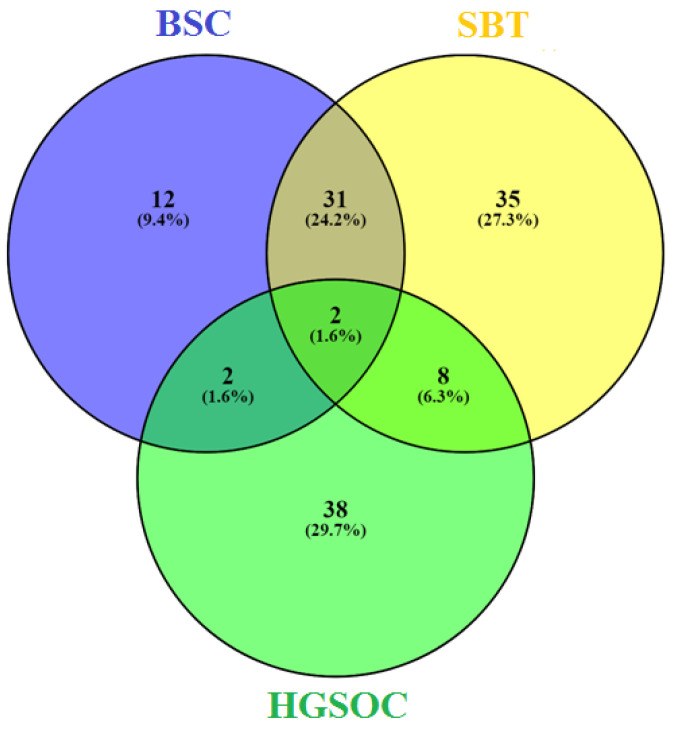
Venn diagram of differentially expressed miRNAs in serous ovarian tumors.

When comparing the miRNA read counts in the analyzed samples of ovarian tumors with those in samples of histologically normal fallopian tube fimbriae, the lists of differentially expressed miRNAs were obtained for each type of tumor, namely, for BSC (Table S1, Sheet 1), for SBT (Table S1, Sheet 2) and for HGSOC (Table S1, Sheet 3). When comparing identified miRNA signatures for each kind of ovarian serous tumor by constructing a Venn diagram, the similarity of the BSC and SBT was revealed, but molecular biological profiles of both kinds of tumors, BSC and SBT, were almost completely different from that of HGSOC (Figure 2).

The obtained data confirm the hypothesis of two different pathogenetic mechanisms for the formation of serous ovarian tumors: a common mechanism for BSC and SBT, and another mechanism for HGSOC (see the Introduction section). The list of miRNAs that significantly differentiated BSC and SBT from HGSOC is presented in Table S1, Sheet 4. To validate the NGS data by quantitative RT-PCR, we randomly selected eight miRNAs from Table S1, Sheet 4, namely: hsa-miR-17-5p, hsa-miR-425-5p, hsa-miR-20a-5p, hsa-miR-93-5p, hsa-miR-30d-5p and hsa-miR-16-5p, with a high level of expression in HGSOC relative to BSC and SBT, and hsa-miR-101-3p and hsa-miR-140-3p, with a lower level of expression in HGSOC relative to BSC and SBT (Table 3).

### 3.2. Validation of NGS Data by Quantitative RT-PCR

**Figure 3:**
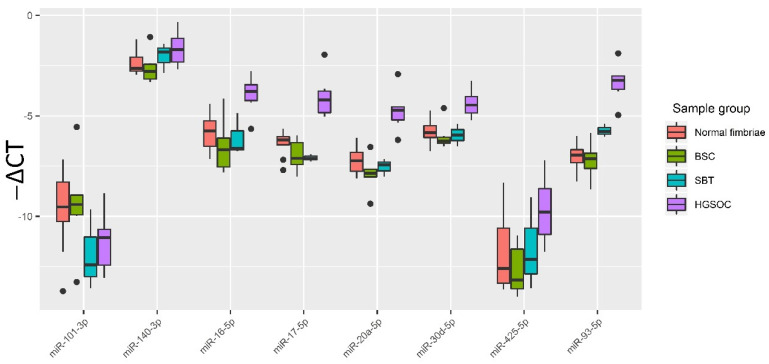
Quantitative RT-PCR data on the miRNA expression level (−∆Ct values) in the tissue samples from patient cohort 1. Data are presented as the median of the “−∆Ct” values, quartiles Q1 and Q3, and outliers as the dots.

The relative expression of hsa-miR-17-5p, hsa-miR-425-5p, hsa-miR-20a-5p, hsa-miR-93-5p, hsa-miR-30d-5p, hsa-miR-16-5p, hsa-miR-101-3p and hsa-miR-140-3p in all samples from Table 1 (normal fimbriae, n = 9; BSC, n = 6; SBT, n = 3; HGSOC, n = 6) was determined by the ∆Ct method using SNORD68 as the reference RNA. To visualize the data obtained, a box diagram was plotted (Figure 3).

A two-tailed Wilcoxon–Mann–Whitney test was used to evaluate the significance of the differences of the matched groups by miRNA expression level (-∆Ct values), and the data are presented in Table 3. From Table 4 and Figure 3, it follows that, in HGSOC, the expression levels of miR-16-5p, miR-17-5p, miR-20a-5p, miR-93-5p and miR-30d-5p are statistically significantly elevated compared to normal tubal fimbriae or BSC or SBT, which is consistent with NGS data (Table S1, Sheet 1–4).

### 3.3. Evaluation of the Diagnostic Potential of miR-16-5p, miR-17-5p, miR-20a-5p, miR-93-5p and miR-30d-5p, Circulating in the Peripheral Blood Plasma of Patients with HGSOC

The expression level of miR-16-5p, miR-17-5p, miR-20a-5p, miR-93-5p and miR-30d-5p, which are tissue-specific for serous ovarian tumors and statistically significantly differentiate HGSOC from BSC and SBT, was analyzed by quantitative RT-PCR in peripheral blood plasma of the second cohort of patients included in the following groups: 1 control group of 13 patients aged 33 to 54-years-old (average level of CA125 was equal to 16.5 U/mL in the range from 9.7 to 42.0 U/mL); 2 control groups of 13 patients aged 25 to 33-years-old (average level of CA125 was equal to 11 U/mL in the range from 2 to 19 U/mL); 20 HGSOC patients aged 33 to 77-years-old (average level of CA125 was equal to 791 U/mL in the range from 30 to 3808 U/mL); 12 BSC patients aged 34 to 64 (average level of CA125 was equal to 18 U/mL in the range from 4.8 to 31.2 U/mL); 18 SBT patients aged 27 to 52-years-old (average level of CA125 was equal to 32 U/mL in the range from 2.9 to 143 U/mL); 10 LGSOC patients aged 27 to 54-years-old (average level of CA125 was equal to 324.6 U/mL in the range from 56.3 to 603.9 U/mL); 11 patients with ovarian endometrioma aged 33–47-years-old (average level of CA125 was equal to 34.9 U/mL in the range from 14.8 to 52 U/mL); 12 patients with deep infiltrating endometriosis aged 28–42-years-old (average level of CA125 was equal to 45.6 U/mL in the range from 26.2 to 65 U/mL). miR-30d-5p was used as an endogenous control RNA due to minor deviations in the level in the blood plasma (standard deviation is 0.25) in the entire second cohort of patients.

**Figure 4:**
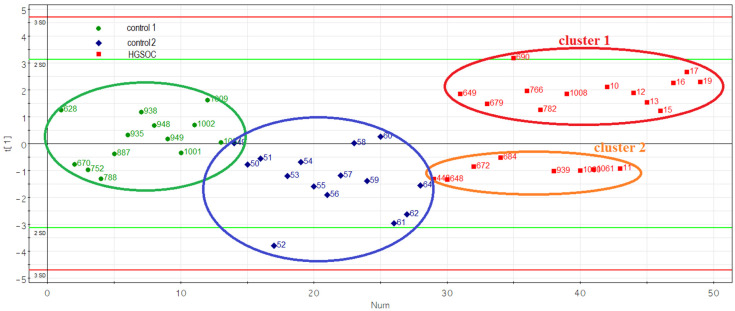
Principal component analysis (PCA) plot based on the miRNA dataset in the control and HGSOC groups.

Principal component analysis of the miR-16-5p, miR-17-5p, miR-20a-5p and miR-93-5p levels in two control and HGSOC groups revealed the formation of two clusters of HGSOC samples (Figure 4).

The detailed characteristics of 20 HGSOC patients are presented in Table 5.

**Figure 5:**
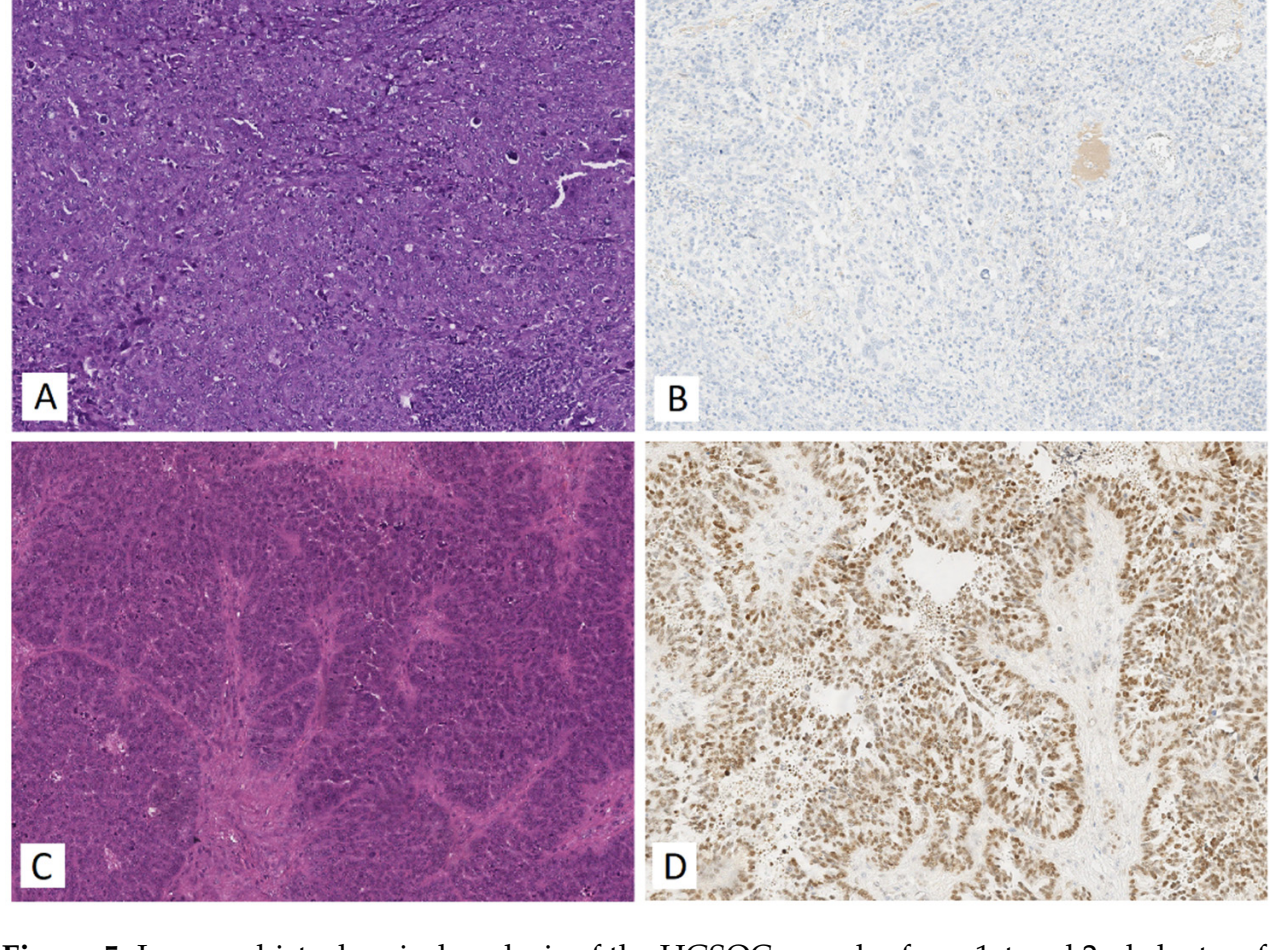
Immunohistochemical analysis of the HGSOC samples from 1st and 2nd clusters formed by PCA in Figure 4. (**A**) Hematoxylin and eosin and (**B**) anti-progesterone receptor antibody staining of 1st cluster tumor (sample ID 19); (**C**) hematoxylin and eosin and (**D**) anti-progesterone receptor antibody staining of the 2nd cluster tumor (sample ID 672).

Samples from cluster 1 and cluster 2 (Figure 4) were statistically significantly different in RECIST 1.1 MRI/CT criteria (*p* = 0.0191): pre-surgery CA 125 level (*p* = 0.05), tumor size (tumor length, *p* = 0.0114; tumor height, *p* = 0.0192; tumor width, *p* = 0.0483), surgery time (*p* = 0.013), surgery blood loss (*p* = 0.0175), hsa-miR-16-5p miRNA expression level (*p* = 0.0001), hsa-miR-17-5p (*p* = 0.0001), hsa-miR-20a-5p (*p* < 0.0001) and hsa-miR-93-5p (*p* < 0.0001). When analyzing the expression level of the progesterone receptor in HGSOC tissues, a negative immunohistochemical reaction was detected in 12 out of 13 samples (92.3%) of cluster 1 HGSOC, while a positive immunohistochemical reaction was detected in 5 out of 7 samples (71.4%) of cluster 2 HGSOC. Representative images of the immunohistochemical staining of HGSOC tissue sections from clusters 1 and 2 are shown in Figure 5.

Spearman correlation analysis revealed that the expression level of the progesterone receptor in the HGSOC tissue was statistically significantly inversely correlated with the level of miR-17-5p (r = −0.46, *p* = 0.043) and miR-16-5p (r = −0.49, *p* = 0.0282), which in turn were inversely correlated with tumor size (length: r = −0.58 and *p* = 0.0069 for miR-17-5p, r = −0.75 and *p* = 0.0002 for miR-16-5p; width: r = −0.64 and *p* = 0.0022 for miR-17-5p, r = −0.67 and *p* = 0.0012 for miR-16-5p; height: r = −0.7 and *p* = 0.0006 for miR-16-5p, r = −0.7 and *p* = 0.0006 for miR-16-5p), surgery time (r = −0.63 and *p* = 0.0035 for miR-17-5p, r = −0.59 and *p* = 0.0076 for miR-16-5p), surgery blood loss (r = −0.55 and *p* = 0.0119 for miR-17-5p, r = −0.49 and *p* = 0.0294 for miR-16-5p). Eight out of thirteen (61.5%) patients with HGSOC (cluster 1) underwent suboptimal cytoreduction, and in the remaining cases (38.5%), complete cytoreduction was conducted (Table 5). In contrast, five out of seven patients (71.4%) with HGSOC (cluster 2) underwent a complete cytoreduction and only 28.6% of patients underwent suboptimal cytoreduction (Table 5). According to the RECIST 1.1 criteria, three out of thirteen patients (23%) with HGSOC (cluster 1) showed a complete tumor response to ongoing chemotherapy (carboplatin AUC 6 + paclitaxel 175 mg/m2); in 7 out of 13 cases (54%), there was a stabilization of the condition, and in 3 out of 13 cases (23%), there was a progression of the disease (Table 5). Among patients with HGSOC (cluster 2), there was a complete tumor response to chemotherapy in six out of seven cases (85.7%) and disease progression in one out of seven cases (14.3%).

**Figure 6:**
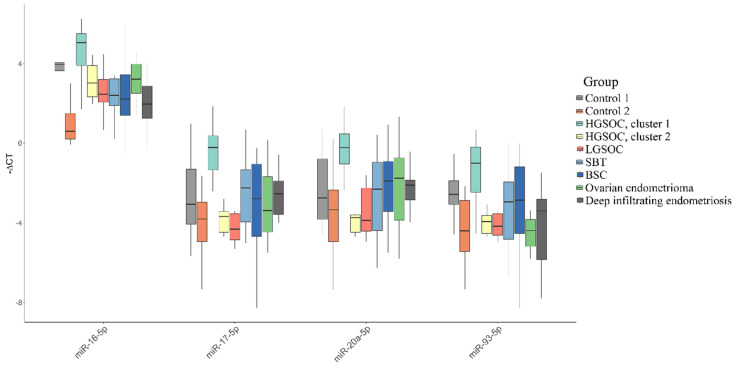
Comparative analysis of miRNA expression level in blood plasma samples of apparently healthy women, patients with different histotypes of serous ovarian tumors, endometriosis by quantitative real-time PCR. Data are presented as the median of the “-∆Ct” values, quartiles Q1 and Q3, and outliers as the dots.

The “-ΔCt” values in two HGSOC molecular subtypes were compared with those in two control groups (control 1, control 2), as well as with groups of women with other serous ovarian tumors and groups of women with endometriosis. In spite of the fact that only ovarian clear cell carcinoma and endometrioid ovarian cancer have links to endometriosis, groups of women with ovarian endometriosis and deep infiltrating endometriosis were included in this study as a variant of the female reproductive organ diseases. Furthermore, the endometrium is derived from the intermediate mesoderm via mesenchymal-to-epithelial transition during the development of the urogenital system, and during the pathogenesis of deep infiltrating endometriosis, endometrial epithelial cells may be prone to return to this state via epithelial-to-mesenchymal transition (EMT) [40]. In light of the above, we aimed to compare the blood plasma of patients with HGSOC and pelvic endometriosis by the quantitation of the miR-16-5p, miR-17-5p, miR-20a-5p and miR-93-5p as the key molecules in the EMT (see Section 3.5). The results of the comparison are presented in the form of box diagrams in Figure 6, Table 6, with indication of the “-ΔCt” median values and Q1 and Q3 quartiles, and Table 7, with indication of the significance of differences between the compared groups using a two-sided Wilcoxon–Mann–Whitney test. It was found that miR-16-5p, miR-17-5p, miR-20a-5p and miR-93-5p significantly differentiated the HGSOC of the cluster 1 group from the following groups: (i) HGSOC of cluster 2, (ii) LGSOC, (iii) SBT, (iv) BSC, (v) ovarian endometrioma and (vi) deep infiltrating endometriosis. At the same time, in comparison with the control groups, which differed in the patients’ age (control 1, 33–54-years-old; control 2, 25–33-years-old), the level of expression of all four miRNAs was significantly higher in the group of patients with HGSOC of cluster 1 than in all other groups of patients with serous tumors and groups of patients with endometriosis. Importantly, the two control groups differed statistically significantly in their levels of circulating miR-93-5p (*p* = 0.0096) and miR-16-5p (*p* < 0.0001), with elevated levels of their expression in the older group. On the contrary, no dependence of miR-17-5p and miR-20a-5p levels in peripheral blood plasma on the age of the patients was found, and these two miRNAs were specific plasma markers of progesterone receptor-negative HGSOC (cluster 1), statistically significantly differentiating patients with this subtype of HGSOC from apparently healthy women and patients with other types of serous ovarian tumors or external genital endometriosis. In the blood plasma of patients with HGSOC, highly expressing the progesterone receptor, a statistically significant decrease in the level of miR-93-5p was found, in contrast to patients with a progesterone receptor-negative HGSOC tissue, in whose blood plasma a statistically significant increase in the level of miR-93-5p was detected, when compared with control group 1. A statistically significant decrease in the level of miR-93-5p was also found in the blood plasma of patients with endometrioid ovarian cysts when compared with control group 1, so this miRNA cannot be considered a unique HGSOC marker.

### 3.4. Evaluation of the Prognostic Potential of miR-16-5p, miR-17-5p, miR-20a-5p and miR-93-5p, Circulating in the Peripheral Blood Plasma of Patients with HGSOC

**Figure 7:**
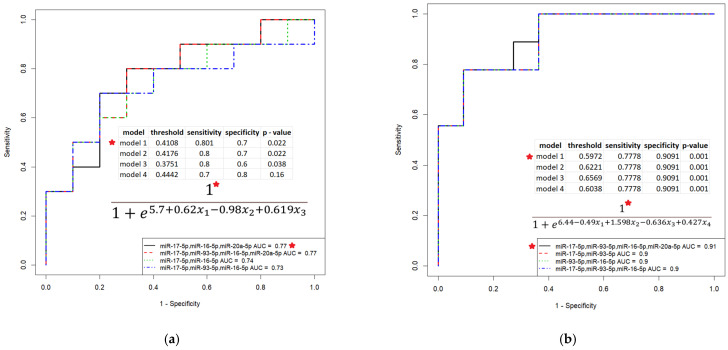
Models of logistic regression for HGSOC patients based on the ∆Ct values for the miR-16-5p, miR-17-5p, miR-20a-5p and miR-93-5p in the peripheral blood plasma. (**a**) Prediction for complete cytoreduction; (**b**) Prediction for complete response to post surgery chemotherapy. For the combination of miRNA molecules indicated by a red asterisk, the parameters and formula of the logistic regression model are given.

In light of the above, the possible link between the level of circulating miR-16-5p, miR-17-5p, miR-20a-5p and miR-93-5p in the blood of HGSOC patients with surgical outcome and post surgery response to chemotherapy was evaluated. This analysis of the data is relevant, since there is no clinically applicable biomarker that can predict suboptimal cytoreduction, which is associated with poor overall survival, as discussed in a number of systematic reviews [41,42,43]. To develop prediction models of logistic regression, clinical characteristics of the HGSOC patients from Table 4 (surgical outcome—complete or suboptimal cytoreduction; RECIST 1.1 data—complete response or resistance to chemotherapy in the case of partial response, stable disease and progressive disease) and -∆Ct values for each of the miR-16-5p, miR-17-5p, miR-20a-5p and miR-93-5p were used. The developed models are presented in Figure 7.

It was found that the combined determination of the level of circulating miR-16-5p, miR-17-5p, miR-20a-5p and miR-93-5p circulating in blood plasma in patients with primary HGSOC tumors makes it possible to predict the optimal cytoreduction with 80.1% sensitivity and 70% specificity (Figure 7a, model 1: *p* = 0.022, TPR = 0.8, FPR = 0.3, probability of optimal cytoreduction at calculated values according to formula of the model > 0.4108) and the complete response to post surgery chemotherapy with 77.8% sensitivity and 90.9% specificity (Figure 7b, model 1: *p* = 0.001, TPR = 0.78, FPR = 0.09, probability of chemosensitivity at calculated values according to formula of the model > 0.5972). The use of these models can predict cases where complete cytoreduction cannot be achieved due to difficulty in resecting tumors that have invaded vital organs. In such cases it would be preferable to forego primary cytoreduction surgery and use neoadjuvant chemotherapy to reduce the residual tumor mass and increase the chances of achieving complete interval cytoreduction. For instance, the addition of hyperthermic intraperitoneal chemotherapy to interval cytoreductive surgery after three cycles of neoadjuvant chemotherapy with carboplatin and paclitaxel resulted in a 12-month increase in overall survival for patients who were not eligible for an initial macroscopic complete resection [44].

### 3.5. Functional Significance of miR-16-5p, miR-17-5p, miR-20a-5p and miR-93-5p in Determination of Different HGSOC Subtypes

**Figure 8:**
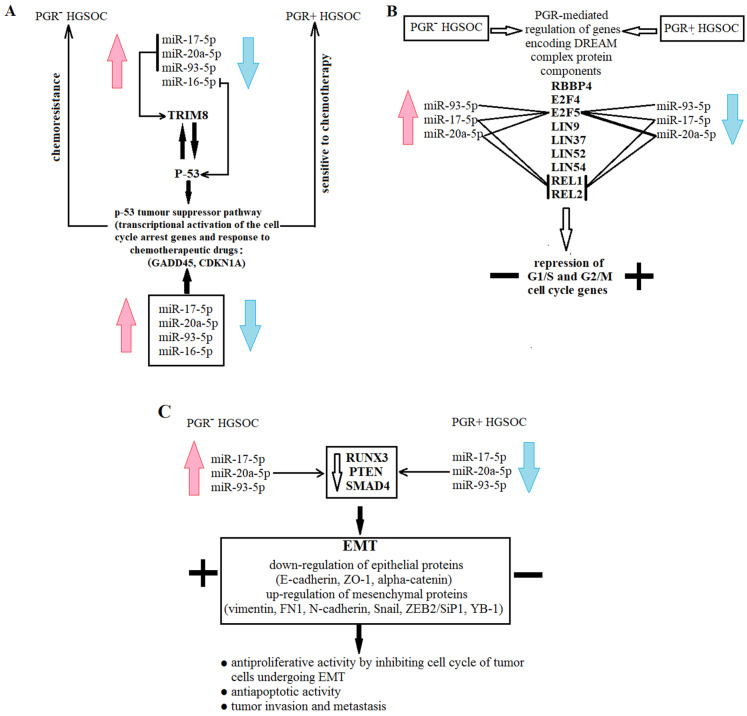
Scheme of functional significance of miR-16-5p, miR-17-5p, miR-20a-5p and miR-93-5p. Schematic participation of miRNAs in chemoresistance (**A**), cell proliferation (**B**), and EMT (**C**) is presented in the form of different blocks.

Using the MiRTargetLink database (https://ccb-compute.cs.uni-saarland.de/mirtargetlink2/bidirectional_search/) (accessed on 30 August 2022), we focused our attention on those miRNA target genes that can determine the proliferative, invasive and metastatic properties of primary HGSOC tumors, which in turn determine the success of cytoreduction and sensitivity to postoperative chemotherapy (Figure 8).

According to this database, miR-17-5p, miR-20a-5p and miR-93-5p negatively regulate TRIM8, which is a direct target of the *P53* gene [45]—the main participant in the p53 tumor suppressor pathway [46,47,48]. In turn, the p53 protein level is controlled by miR-16-5p (MiRTargetLink data) and by TRIM8, aninducer of the degradation of the MDM2 protein, which is the principal negative regulator of p53 stability [45]. TRIM8 deficit, in particular due to miR-17-5p upregulation, contributes to the impairment of p53-mediated responses to chemotherapeutic drugs and results in chemoresistance and oncogenesis [49]. Moreover, miR-17-5p may perform a role in the development of drug resistance in cancer cells by targeting the anti-apoptotic p21 protein (CDKN1A) [50]. Therefore, there is a feedback loop with the participation of p53, TRIM8 and miRNAs, which control their protein levels: p53 promotes the transcription of TRIM8, which, in turn, interacting with p53, induces its stabilization and the p53-dependent transcriptional activation of cell cycle arrest genes, such as *CDKN1A* and *GADD45*, and this axis is under the control of miR-17-5p, miR-20a-5p, miR-93-5p and miR-16-5p (according to MiRTargetLink data) in defining drug responsiveness and cell proliferation (Figure 8a).

The two molecular subtypes of HGSOC that we detected differed in the expression level of the progesterone receptor (PGR-negative and PGR-positive), and the PGR-negative phenotype of HGSOC was associated with the upregulation of miR-17-5p, miR-20a-5p, miR-93-5p and miR-16-5p. Although these miRNAs do not directly regulate the level of the PGR, their experimentally proven target genes are transcription factors binding to promoter/enhancer sites of the PGR gene, according to the MiRTargetLink database; in particular: miR-17-5p downregulates *PKNOX1, CBX8, MNT*; miR-16-5p—*POLR2A, TBP, SMARCA4, NFIC, PKNOX1, ZFX*; miR-93-5p—*PKNOX1, CBX8, ZIC2, POLR2A, EZH2*; miR-20a-5p—*PKNOX1, CBX8*. The common target for all four miRNAs is the homeobox protein *PKNOX1* gene. The action of progesterone through the PGR mediates the transcriptional regulation of DREAM complex proteins supporting the DREAM/DYRK1-mediated repression of cell cycle-dependent genes [51]. According to the MiRTargetLink data, the expression level of some of the DREAM complex proteins, namely, E2F5, REL1 and REL2, are under the control of the miR-17-5p, miR-20a-5p and miR-93-5p (Figure 8b), providing the proliferative potential and survival of the tumor cells.

The experimentally proven target genes of miR-17-5p, miR-20a-5p and miR-93-5p (from MiRTargetLink data base) are *RUNX3, PTEN* and *SMAD4*, which cause the activation of the downstream EMT signaling in the case of their decreased expression level (Figure 8c); in particular: upregulation of mesenchymal proteins—vimentin, FN1, N-cadherin, Snail, ZEB2/SiP1, YB-1, and downregulation of epithelial proteins—E-cadherin, ZO-1, α-catenin [52,53,54,55,56,57,58,59,60]. It was found that cells undergoing EMT are characterized by the antiproliferative properties with high invasiveness, metastatic ability, resistance to apoptosis, radio- and chemotherapy [61,62,63]. So it was suggested that high cell proliferation is necessary for the initial stages of the pathogenesis of primary tumor and its maintenance, while inhibition of cell division is a key step for ensuring the invasion and migration of tumor cells [58].

## 4. Discussion

Ovarian cancer incidence and mortality cases increased by 88.01 and 84.20%, respectively, from 1990 to 2017, all over the world [64]. Serous ovarian cancer accounts for 75–80% of ovarian malignancies. A better understanding of the pathogenesis and molecular classification of ovary cancer is urgently needed because the mortality rate has remained high and five-year survival rates are low; in particular, for the dominant tumor histologic type in epithelial ovarian cancers, HGSOC, these values are approximately equal to 40.6% [65] or to 70–80% [66].

In the present study, we focused on the identification of the miRNA signature of HGSOC tissue in comparison with other serous ovarian tumors (BSC and SBT) by deep sequencing, followed by the validation of selected miRNAs by real-time quantitative PCR. An analysis of differentially expressed miRNAs in ovarian tumor tissues relative to normal fallopian tube fimbriae revealed scant similarity between HGSOC and BSC (3.2% of 128 analyzed miRNAs) and SBT (7.9%), but a greater similarity between BSC and SBT (33%). The hierarchical clustering of miRNA profiles of tumor tissues demonstrated the formation of a separate cluster of HGSOC samples and a common cluster of BSC and SBT samples. The data obtained confirm the dualistic classification of epithelial ovarian cancers based on the differences in morphological types with specific molecular changes and differences in prognosis [67]: type I ovarian neoplasms are usually low-grade tumors without *TP53* mutations, with slow progression and with a good prognosis if they are diagnosed in the early stages (LGSOC, mucinous carcinomas, clear cell carcinomas, and endometroid ovarian carcinomas), and type II ovarian neoplasms (HGSOC), which are characterized by mutations of *TP53* and frequently diagnosed in the advanced stages with a poor prognosis. The precursor lesion of HGSOC is considered to be serous tubal intraepithelial carcinomas (STICs), which was proved by revealing identical somatic *TP53* mutations in STICs and concurrent HGSOCs [68] and the identification of STICs in 11–61% of cases with HGSOC [69]. As for LGSOC, it is thought to progress from BSC in a stepwise fashion via SBT, which was confirmed by an in vitro carcinogenic model [12] and by genetic analyses of the serous cystadenomas [70]. Another hypothesis is that a papillary tubal hyperplasia (PTH) is the source of origin for LGSOC. According to this hypothesis, the PTH, as a precancerous lesion, develops in the fallopian tube and involves the ovary secondary having the same morphology as an SBT (papillary structures, branching, psammoma bodies and salpingoliths) [71,72,73]. Consequently, only the progression from an SBT to LGSOC occurs in the ovary.

Two subtypes of HGSOC were identified, which significantly differ in the level of expression of the progesterone receptor in the tumor tissue, the secretion of miR-16-5p, miR-17-5p, miR-93-5p and miR-20a-5p, and the level of serum CA125, tumor size, surgical outcome (optimal or suboptimal cytoreduction) and response to chemotherapy. In particular, we identified patients with the progesterone receptor-negative subtype of HGSOC, characterized by an increased level of miR-16-5p, miR-17-5p, miR-93-5p and miR-20a-5p in peripheral blood, by lower serum CA-125 levels, smaller tumor size, suboptimal cytoreduction in 61.5% of cases and a complete response to adjuvant chemotherapy in only 20% of cases, in comparison with the progesterone receptor-positive subtype of HGSOC, with optimal cytoreduction in 71.4% of cases and a complete response to adjuvant chemotherapy in 85.7% of cases. It was found here that the combined determination of the level of circulating miR-16-5p, miR-17-5p, miR-20a-5p and miR-93-5p circulating in blood plasma in patients with primary HGSOC tumors makes it possible to predict the optimal cytoreduction with 80.1% sensitivity and 70% specificity; furthermore, the combination of miRNAs can predict a complete response to post surgery chemotherapy with 77.8% sensitivity and 90.9% specificity. It is assumed that the success of cytoreductive surgery, as well as response to chemotherapy, are dictated by EOC biology, and the low probability of optimal cytoreduction is associated with the progression of the disease and poor overall survival [41,42,74]. While comparing primary tumors from optimally and suboptimally cytoreduced patients, Liu Z. and colleagues [74] revealed the gene network associated with increased stromal activation and lymphovascular invasion of a distinct mesenchymal molecular subtype of EOC. This molecular signature (*POSTN, FAP, TIMP3, CTSK, TNFAIP6, CXCL14, FAP, TIMP3* and *COL11A1*) is overlapped with the gene signature of suboptimal debulking identified by Riester M. [75] and by Tucker S. [76], among which the expression of *POSTN, FAP* and *TIMP3* was associated with therapeutic resistance in EOC [77].

The main regulators of the expression of key genes involved in the pathogenesis of certain molecular subtypes of serous ovarian cancer are small non-coding RNAs, including miRNAs. These small molecules could be used as diagnostic and prognostic molecules [78], as they regulate multiple pathways implicated in cell proliferation, differentiation, cell migration and apoptosis [79,80], by targeting mRNAs and repressing translation as a part of the RISC [81,82]. Kuznetsov VA and colleagues, using big-data analytics, identified 19 miRNAs and 31 miRNAs expressed in tumors as prognostic classifiers, allowing the separation of the HGSOC patients into low-, intermediate- and high-risk subgroups with a five-year survival rate of 51.6–85%, 20–38.1% and 0–10%, respectively, which were also correlated with post surgery chemotherapy response [83]. In particular, low- and high-risk patients were significantly correlated with the “proliferative, sensitive to chemotherapy” and “mesenchymal, chemoresistance” subtypes of HGSOC, respectively. It has been established that it is rapidly dividing cells that are sensitive to chemotherapy, which explains the resistance to chemotherapy of a mesenchymal-type tumor with signs of stemness, in contrast to a proliferative-type tumor with a good response to chemotherapy [66,84,85,86,87]. In the present study, we divided the HGSOC samples into two subtypes according to the content of miR-16-5p (from mir-15 family), miR-17-5p and miR-20a-5p (from the miR-17/92 cluster of the mir-17 family), and miR-93-5p (from the miR-106b/25 cluster of the mir-17 family) circulating in the blood plasma of patients. The participation of these miRNAs in the induction of EMT was revealed, in particular, by targeting *RUNX3* [52], by the silencing of *CYB7B1* [88], through the PTEN/Akt pathway [59,89] and by downregulating Smad4 [55].

Our data are in good agreement with those of other colleagues who showed increased levels of miR-20a-5p, miR-16-5p and miR-93-5p in serous ovarian cancer tissues compared with the corresponding normal tissues [28,90], upregulated miR-16-5p and miR-17-5p in the peripheral blood lymphocytes of patients with ovarian cancer [91], and upregulated miR-93-5p in the serum of patients with SOC [92]. The overexpression of miR-19a and miR-19b-1, the key oncogenic components of the miR-17-92 cluster, triggers the EMT of lung cancer cells, providing cancer invasion and metastatic dissemination, the resistance of tumor cells to radiotherapy and chemotherapy, and apoptosis, associated with poor prognosis of cancer patients [58]. The authors of this article underline that the upregulation of cell proliferation is important for the initiation and maintenance of primary tumors, but during EMT, the proliferative rates of tumor cells decrease to enable them to reach their new destinations.

The identification of miR-16-5p, miR-17-5p, miR-20a-5p and miR-93-5p in blood plasma, elevated in suboptimally versus optimally cytoreduced patients, their increased HGSOC tissue expression level and the association with the mesenchymal tumor type make it reasonable to speculate that a changed level of these miRNAs in blood plasma reflects the biological characteristics of the tumor tissue itself. Jaynish S. Shah with colleagues proposed a combination of miR-34a-5p and CA125 levels to classify women with an optimal or suboptimal cytoreduction with an AUC of 0.818 and an accuracy of 0.786 [93]. However, according to the authors of the study themselves, the elevated miR-34a-5p in the blood plasma of suboptimally cytoreduced patients did not originate from the tumor tissue itself but may reflect a systemic inflammatory response to the pattern of spread of HGSOC. Consistent with our NGS data, miR-34a-5p did not significantly differentiate BSC and SBT from HGSOC (*p* = 0.449, data are not shown). That is why we did not validate this miRNA by RT-PCR in tissue and plasma samples.

We also observed statistically significant differences between the two molecular subtypes of HGSOC in terms of tumor size, where a smaller tumor volume was characteristic of progesterone receptor-negative HGSOC, with increased levels of extracellular miR-16-5p, miR-17-5p, miR-93-5p and miR-20a-5p, and with more aggressive tumor behavior responsible for the failure of optimal surgical resection in most cases. In addition, in our study, HGSOC patient groups (PGR-positive and PGR-negative tumors) significantly differed by the serum CA125 levels, with a more pronounced increase in CA125 in the group of patients with a high level of expression of the PGR in the tumor. Our data reflect the proven relationship between CA125 and tumor cell invasiveness through binding with E-cadherin and β-catenin complexes, and in the case of the downregulation of cell-surface CA125/MUC16, EMT is promoted [94]. In light of this fact, it is not excluded that the possibility of increased EMT under decreased CA125 and the upregulation of miR-16-5p, miR-17-5p, miR-93-5p and miR-20a-5p in the case of PGR-negative HGSOC results in a smaller tumor size, unresectable tumor and worse survival compared to PGR-positive HGSOC. According to a systematic review and meta-analysis, the high expression of circulating miR-20a is a risk factor for unfavorable prognosis for patients with cancers [95]. It was demonstrated that an increased expression of miR-20a could promote the activation of the NFκB pathway by targeting *NFKBIB* (alternative name IκBβ) and result in the increased expression of p65, livin and survivin, which potentially contribute to a decrease in the gastric cancer cell apoptosis induced by cisplatin and chemoresistance [96]. The other members of the miR-17/92 cluster have also been associated with an unfavorable prognosis and reduced overall survival [97,98,99,100]. In our study, complete cytoreduction and responsiveness to chemotherapy was observed in HGSOC patients with reduced plasma levels of miR-20a-5p and miR-17-5p relative to controls. On the contrary, a pronounced increased level of miR-20a-5p and miR-17-5p in patients with HGSOC in blood plasma relative to the control was associated with the inability to carry out a complete cytoreduction and with an incomplete response to chemotherapy.

In the present study, we found that two molecular subtypes of HGSOC, with increased and decreased levels of secretion miR-16-5p, miR-17-5p, miR-93-5p and miR-20a-5p, are characterized by the absence or presence of progesterone receptor expression, respectively. Moreover, statistically significant inverse correlations of the expression level of the progesterone receptor in the HGSOC tissue with the level of circulating miR-17-5p (r = −0.46, *p* = 0.043) and miR-16-5p (r = −0.49, *p* = 0.0282) were estimated. Although miR-16-5p, miR-17-5p, miR-93-5p and miR-20a-5p do not directly regulate the level of the PGR, their experimentally proven target genes are transcription factors binding to promoter/enhancer sites of the PGR gene, according to the MiRTargetLink database. In addition, the common target for all four miRNAs is found to be the homeobox protein *PKNOX1* gene. *PKNOX1* (alternative name is *PREP1*) is a tumor suppressor gene that is linked to the definition of DNA replication timing of a significant portion of the genome and prevents DNA damage [101]. Unlike normal human tissues expressing PREP1, the vast majority of human cancers lack PREP1 and are characterized by genomic instability and DNA damage [102,103]. Thus, the elevated secretion of miR-17-5p, miR-20a-5p, miR-93-5p and miR-16-5p by HGSOC tissue, associated with high tumor invasiveness and low probability of optimal cytoreduction, may reflect DNA instability and damage in tumor tissue and provide the PGR-negative phenotype of HGSOC.

The PGR has a crucial role in protecting against the occurrence of ovarian cancer by clearance of p53-defective lesions through the TNF-a/RIPK1/RIPK3/MLKL pathway, inducing cell necroptosis [104]. It is believed that HGSOC, in contrast to other ovarian cancer subtypes, originates predominantly from serous tubal intraepithelial carcinomas (STICs) possessing TP53 mutations and showing little to no proliferative activity under the PGR regulation of DREAM complex genes, repressing 900 cell cycle genes [51]. Whether the transition from “dormant” STICs with low proliferation to “active” STICs with high proliferation, able to shed and disseminate, is accompanied by a change in PGR signaling is not known. However, according to the western blot analyses carried out by Mauro L.J. and colleagues, all analyzed normal human fallopian tube tissues have a robust expression of both PGR isoforms; PGR staining was observed in STICs, and this expression was retained in invasive HGSC tissue, but only ~35% of metastatic HGSC tumors expressed abundant progesterone receptors [51]. This is consistent with the data of Tone A.A. et al. [105], which demonstrated a strong downregulation of PGR expression in HGSOC, with >10% positivity for PR-A and PR-B in 20% and 25% of all HGSOC cases, respectively. In our HGSOC patient cohort, 6/20 (30%) showed an Allred score ≥ 3 and were considered positive for PGR-A/B. Since the level of the PGR expression was found to be different in the two subtypes of HGSOC we identified, and PGR level was associated with a certain miRNA signature, the possibility of optimal cytoreduction, and sensitivity to chemotherapy, larger studies are needed to confirm/refute these relationships.

## 5. Conclusions

To date, there is no clinically applicable biomarker that can predict suboptimal cytoreduction. The search for such biomarkers is very important for the HGSOC patient because, in the case where primary complete cytoreduction cannot be achieved due to difficulty in resecting tumors that have invaded vital organs, patients are more likely to benefit from neoadjuvant chemotherapy to reduce the tumor burden and increase the chances of achieving complete interval cytoreduction surgery. We found that the combined determination of the level of circulating miR-16-5p, miR-17-5p, miR-20a-5p and miR-93-5p circulating in blood plasma in patients with primary HGSOC tumors makes it possible to predict optimal cytoreduction with 80.1% sensitivity and 70% specificity (*p* = 0.022, TPR = 0.8, FPR = 0.3). Closer examination of the molecules, in particular, circulating miRNAs, that have already been identified in association with the mesenchymal subtype of HGSOC and poor overall survival [7,106,107,108,109], in addition to our results, may reveal important information about the biology of unresectable HGSOC and can make a significant contribution to the creation of a test system for predicting suboptimal cytoreduction.

## Figures and Tables

**Table 1:** miRNA parameters.

miRNA	miRNA Accession Number (miRBase), available online: http://www.mirbase.org/ (accessed on 15 August 2022)	Nucleotide Sequence of Sense Primer for PCR, 5’-3’	PCR Primer Annealing Temperature, °C
hsa-miR-16-5p	MIMAT0000069	TAGCAGCACGTAAATATTGGCG	62
hsa-miR-17-5p	MIMAT0000070	CAAAGTGCTTACAGTGCAGGTAG	55
hsa-miR-20a-5p	MIMAT0000075	TAAAGTGCTTATAGTGCAGGTAG	52
hsa-miR-93-5p	MIMAT0000093	CAAAGTGCTGTTCGTGCAGGTAG	55
hsa-miR-425-5p	MIMAT0003393	AATGACACGATCACTCCCGTTGA	60
hsa-miR-101-3p	MIMAT0000099	TACAGTACTGTGATAACTGAA	55
hsa-miR-140-3p	MIMAT0004597	TACCACAGGGTAGAACCACGG	55
hsa-miR-30d-5p	MIMAT0000245	TGTAAACATCCCCGACTGGAAG	55

**Table 2:** Sample characteristics of patient cohort 1.

Sample ID	Sample Description	Location in Lower Pelvis	Patient ID	Age	Menstrual Cycle Day	Duration of Menopause, Years	Diagnosis	FIGO [38]	pTNM [39]	NGS	PCR
s26	Normal fimbriae	left side	P1	27	20	0	Endometriosis of the sacro-uterine ligament. Mature cystic teratoma of the left ovary	-	-	Yes	Yes
s27	Normal fimbriae	right side						-	-	No	Yes
s25	Normal fimbriae	right side	P2	30	13	0	Serous cystadenoma of the left ovary, bicornuate uterus with non-functioning closed horn	-	-	Yes	Yes
s11	Normal fimbriae	right side	P3	71	0	21	Right ovarian benign serous cystadenoma	-	-	No	Yes
s12	BSC	right side						-	-	No	Yes
s28	Normal fimbriae	left side	P4	64	0	12	Right ovarian benign serous cystadenoma	-	-	No	Yes
s29	Normal fimbriae	right side						-	-	No	Yes
s30	BSC	right side						-	-	Yes	Yes
s5	Normal fimbriae	left side	P5	77	0	22	Benign serous cystadenomas of both ovaries	-	-	No	Yes
s7	Normal fimbriae	right side						-	-	No	Yes
s6	BSC	left side						-	-	Yes	Yes
s8	BSC	right side						-	-	Yes	Yes
s15	Normal fimbriae	right side	P6	45	28	0	Right ovarian benign serous cystadenoma	-	-	Yes	Yes
s16	BSC	right side						-	-	No	Yes
s4	BSC	right side	P7	69	0	19	Benign serous cystadenomas of both ovaries	-	-	No	Yes
s2	SBT	right side	P8	51	0	2	Borderline serous papillary cystadenoma of the right ovary. Multiple myoma of the uterine corpus. Adenomyosis.	Ic1	pT1c1N0M0	Yes	Yes
s10	SBT	left side	P9	29	12	0	Borderline serous papillary cystadenoma of the left ovary.	Ia	pT1aCN0M0	Yes	Yes
s24	SBT	right side	P10	27	7	0	Borderline serous papillary cystadenoma of the right ovary.	IIa	pT2aCN0M0	Yes	Yes
s18	HGSOC	right side	P11	45	0	1	High-grade serous carcinoma of the right ovary.	IIa	pT2aCN0M0	Yes	Yes
s21	HGSOC	right side	P12	58	0	10	High-grade serous carcinoma of the right ovary. Ascites. Adhesive process in the abdominal cavity.	IIIa2	pT3aCN0M0	Yes	Yes
s14	HGSOC	left side	P13	44	22	0	High-grade serous carcinomas of both ovaries. Small-size uterine myomas.	IIIa2	pT3aCN0M0	No	Yes
s22	HGSOC	right side	P14	53	0	3	High-grade serous carcinoma of the right ovary. Ascites.	IIIa2	pT3aCN0M0	No	Yes
s33	HGSOC	left side	P15	24	0	28	High-grade serous carcinomas of both ovaries. Metastases in the inguinal lymph nodes. Adhesive process in the abdominal cavity. Endometrial polyp.	IVB	pT3aN0M1	No	Yes
s34	HGSOC	right side								Yes	Yes

**Table 3:** NGS data on read counts of miRNAs in serous ovarian tumors.

miRNA	log2 (Fold Change in Expression Level)	lfcSE	*p*-Value *	s6	s8	s30	s2	s24	s10	s18	s21	s34
				BSC			SBT			HGSOC		
hsa-miR-17-5p	3.2	0.5	3.4 × 10^−11^	70.0	56.9	48.6	62.1	61.1	90.4	703.3	294.1	675.4
hsa-miR-425-5p	2.8	0.5	8.9 × 10^−9^	56.6	48.4	75.5	97.6	53.7	119.5	548.0	357.7	316.9
hsa-miR-20a-5p	2.8	0.5	3.4 × 10^−8^	75.8	75.1	73.8	52.8	44.4	65.5	693.2	237.4	607.9
hsa-miR-93-5p	2.7	0.5	1.8 × 10^−7^	386.9	207.7	131.6	366.0	218.3	342.7	1487.4	1260.6	2003.0
hsa-miR-101-3p	−2.5	0.5	2.2 × 10^−7^	989.6	2245.5	2097.1	724.5	1243.3	983.9	353.5	238.5	353.3
hsa-miR-30d-5p	1.4	0.4	1.5 × 10^−4^	14401.1	15283.7	11120.3	14318.1	15006.9	12790.1	38252.0	26761.8	46379.9
hsa-miR-140-3p	−1.9	0.5	4.1 × 10^−4^	1836.6	2932.8	5613.0	2193.9	2908.5	3463.5	717.2	1319.7	709.2
hsa-miR-16-5p	1.9	0.7	6.9 × 10^−3^	36.0	50.3	127.5	60.9	35.2	111.6	116.2	249.9	423.5

* Significance value of differences while comparing HGSOC vs. BSC and SBT.

**Table 4:** Wilcoxon–Mann–Whitney test data on pairwise comparison of normal fimbriae, BSC, SBT and HGSOC groups by miRNA expression level in tissue samples. Significance level (*p*) values are indicated for the corresponding compared groups.

miRNA	Group	Normal Fimbriae	BSC	SBT
miR-16-5p	BSC	0.2238	1	0.7143
miR-16-5p	SBT	0.7273	0.7143	1
miR-16-5p	HGSOC	0.0048	0.0087	0.0476
miR-425-5p	BSC	0.3277	1	0.5476
miR-425-5p	SBT	1	0.5476	1
miR-425-5p	HGSOC	0.0663	0.0152	0.1667
miR-17-5p	BSC	0.2721	1	1
miR-17-5p	SBT	0.1455	1	1
miR-17-5p	HGSOC	0.0004	0.0022	0.0238
miR-20a-5p	BSC	0.3884	1	0.5476
miR-20a-5p	SBT	0.7273	0.5476	1
miR-20a-5p	HGSOC	0.0008	0.0022	0.0238
miR-93-5p	BSC	0.7756	1	0.0476
miR-93-5p	SBT	0.0182	0.0476	1
miR-93-5p	HGSOC	0.0004	0.0022	0.0238
miR-30d-5p	BSC	0.4559	1	0.9048
miR-30d-5p	SBT	0.8636	0.9048	1
miR-30d-5p	HGSOC	0.0016	0.0087	0.0238
miR-140-3p	BSC	0.4559	1	0.3810
miR-140-3p	SBT	0.4818	0.3810	1
miR-140-3p	HGSOC	0.0663	0.0931	0.5476
miR-101-3p	BSC	0.8639	1	0.2619
miR-101-3p	SBT	0.2091	0.2619	1
miR-101-3p	HGSOC	0.1447	0.2403	0.7143

**Table 5:** Sample characteristics of the HGSOC patients.

Sample ID	Age, Years	FIGO	CA 125 Level before Treatment, U/mL	Risk of Malignancy Index (RMI)	Neoadjuvant Chemotherapy	Tumor Length, cm *	Tumor Width, cm *	Tumor Height, cm *	Ascites, mL	Extent of Blood Loss, mL	Surgery Time, min	0—Complete Cytoreduction (Size of Residual Tumor Foci Less than 2.5 mm), 1—Suboptimal Cytoreduction (Size of Residual Tumor Foci 2.5 mm–2.5 cm)	Progesterone Receptor Expression in Tumor, Allred Score **	Cluster Number in Figure 4	RECIST 1.1 MRI/CT Criteria: 1—Complete Response, 2—Partial Response, 3—Stable Disease, 4—Progressive Disease
1008	38	IVB	1244	10,359	No	7	10.5	11	2000	700	480	1	0	1	*3*
766	54	IIB	29	261	No	4	3	3	20	150	140	0	0	1	*1*
690	48	IVB	1340	12,060	No	5.5	3.5	4	50	700	206	0	0	1	*3*
679	51	IIIC	2000	18,000	No	9.5	6	8	1000	750	235	0	0	1	3
649	54	IIIC	200	1800	Yes	*5*	*6*	*7*	3000	650	285	1	0	1	4
15	57	IIC	198	1782	No	18	14	10	1000	400	190	1	0	1	4
19	63	IIIC	41	1206	No	6	2.4	4.6	200	400	265	1	0	1	1
782	45	IIB	517	1551	No	16	16	11.7	50	500	215	0	2	1	3
10	44	IIIB	129	387	No	6	7	6	700	500	175	1	5	1	*1*
13	71	IIIB	517	1551	yes	13	10	8	10	250	165	1	0	1	4
12	45	IIA	60	180	No	4	3	2	10	100	80	0	0	1	3
16	44	IIIC	92	277	No	5	4	4	10	300	185	1	0	1	3
17	47	IA	60	60	No	8.3	7.1	6.6	10	150	151	1	0	1	3
1060	77	IIIC	1203	10,827	No	10	9	8	1200	650	365	0	4	2	*1*
939	33	IIIC	59	270	No	13	12	10	2000	800	205	1	6	2	*1*
672	41	IIIC	1088	3264	No	*9*	*7.5*	*10*	1500	800	275	0	3	2	*1*
684	42	IIIC	1293	3879	No	14	8	6	1500	500	280	0	3	2	*1*
448	51	IVB	3808	11,424	No	15	17	18	10	3000	360	0	0	2	*1*
1061	49	IIIC	1756	5540	No	29.9	15	18.8	9000	3000	590	1	0	2	*1*
11	48	IC	189	567	No	18	10	8	10	300	375	0	6	2	4

* Intraoperative size. ** The Allred score combines the percentage of positive cells and the intensity of the reaction product in most of the carcinomas. Scores of 0–2 are considered negative. Scores of 3–8 are considered positive.

**Table 6:** miRNA expression levels in blood plasma samples of apparently healthy women, patients with various histotypes of ovarian tumors and endometriosis.

	miR-16-5p *	miR-17-5p *	miR-20a-5p *	miR-93-5p *
Control 1	3.96 (−4.06; −3.64)	−3.07 (1.31; 4.08)	−2.76 (0.79; 3.83)	−2.57 (1.88; 3.07)
Control 2	0.59 (−1.5; −0.2)	−3.81 (2.96; 4.94)	−3.34 (2.35; 4.94)	−4.41 (2.87; 5.45)
HGSOC, cluster 1	5.06 (−5.51; −3.91)	−0.21 (−0.37; 1.35)	−0.22 (−0.47; 1.05)	−1.01 (0.19; 2.46)
HGSOC, cluster 2	3.03 (−3.9; −2.33)	−3.69 (3.44; 4.47)	−3.75 (3.59; 4.47)	−3.94 (3.63; 4.53)
LGSOC	2.45 (−3.21; −2.06)	−4.32 (3.54; 4.86)	−3.88 (2.25; 4.42)	−4.17 (3.54; 4.63)
SBT	2.4 (−3.23; −1.89)	−2.24 (1.33; 3.96)	−2.31 (0.97; 4.39)	−2.95 (1.95; 4.83)
BSC	2.22 (−3.45; −1.39)	−2.79 (1.06; 4.69)	−1.89 (0.91; 3.42)	−2.87 (1.2; 4.54)
Ovarian endometrioma	3.22 (−3.98; −2.51)	−3.39 (1.69; 4.46)	−1.76 (0.74; 3.87)	−4.39 (3.84; 5.19)
Deep infiltrating endometriosis	1.97 (−2.87; −1.25)	−2.54 (1.9; 3.58)	−2.11 (1.85; 2.85)	−3.4 (2.81; 5.85)

* Data are presented as median values “-ΔCt”, first and third quartiles, namely: Me (Q1; Q3).

**Table 7:** Results of statistical analysis of miRNA expression levels in blood plasma samples of apparently healthy women, patients with various histotypes of ovarian tumors and endometriosis.

miRNA	Group	Control 1 (33–54 Age) *	Control 2 (25–33 Age) *	HGSOC, Cluster 1 *	HGSOC, Cluster 2 *	LGSOC *	SBT *	BSC *	Ovarian Endometrioma *	Deep Infiltrating Endometriosis *
miR-17-5p	Control 2 (25–33 age)	0.1077	NA	NA	NA	NA	NA	NA	NA	NA
miR-17-5p	HGSOC, cluster 1	0.017	0	NA	NA	NA	NA	NA	NA	NA
miR-17-5p	HGSOC, cluster 2	0.2441	0.9699	0.0024	NA	NA	NA	NA	NA	NA
miR-17-5p	LGSOC	0.1306	0.935	0.0019	0.7925	NA	NA	NA	NA	NA
miR-17-5p	SBT	0.6787	0.0202	0.0224	0.0895	0.0987	NA	NA	NA	NA
miR-17-5p	BSC	0.7689	0.2174	0.0031	0.3355	0.5387	0.5732	NA	NA	NA
miR-17-5p	Ovarian endometrioma	0.649	0.1639	0.0108	0.3502	0.3494	0.412	0.9279	NA	NA
miR-17-5p	Deep infiltrating endometriosis	1	0.0594	0.0031	0.1246	0.2829	0.6615	0.8428	0.8328	NA
miR-93-5p	Control 2 (25–33 age)	0.0096	NA	NA	NA	NA	NA	NA	NA	NA
miR-93-5p	HGSOC, cluster 1	0.0413	2.00 × 10^−4^	NA	NA	NA	NA	NA	NA	NA
miR-93-5p	HGSOC, cluster 2	0.0125	0.9699	0.0062	NA	NA	NA	NA	NA	NA
miR-93-5p	LGSOC	0.0493	0.4952	0.0054	0.9578	NA	NA	NA	NA	NA
miR-93-5p	SBT	0.3732	0.093	0.0106	0.3432	0.7595	NA	NA	NA	NA
miR-93-5p	BSC	0.6495	0.1257	0.0667	0.2496	0.4176	0.8187	NA	NA	NA
miR-93-5p	Ovarian endometrioma	0.0073	0.9188	0.0016	0.6605	0.5116	0.2204	0.1896	NA	NA
miR-93-5p	Deep infiltrating endometriosis	0.0597	0.8667	0.0017	0.4371	0.8212	0.2665	0.2657	0.4865	NA
miR-16-5p	Control 2 (25–33 age)	0	NA	NA	NA	NA	NA	NA	NA	NA
miR-16-5p	HGSOC, cluster 1	0.1184	0	NA	NA	NA	NA	NA	NA	NA
miR-16-5p	HGSOC, cluster 2	0.2441	0.0016	0.0365	NA	NA	NA	NA	NA	NA
miR-16-5p	LGSOC	0.008	0.0192	0.0029	0.4923	NA	NA	NA	NA	NA
miR-16-5p	SBT	0.0073	0.001	0.0066	0.4537	0.9812	NA	NA	NA	NA
miR-16-5p	BSC	0.0457	0.0087	0.0063	0.3845	0.9742	0.7231	NA	NA	NA
miR-16-5p	Ovarian endometrioma	0.0821	6.00 × 10^−4^	0.0037	0.8836	0.1971	0.2962	0.4491	NA	NA
miR-16-5p	Deep infiltrating endometriosis	6.00 × 10^−4^	0.0414	5.00 × 10^−4^	0.0668	0.381	0.3048	0.5512	0.0688	NA
miR-20a-5p	Control 2 (25–33 age)	0.2005	NA	NA	NA	NA	NA	NA	NA	NA
miR-20a-5p	HGSOC, cluster 1	0.0044	1.00 × 10^−4^	NA	NA	NA	NA	NA	NA	NA
miR-20a-5p	HGSOC, cluster 2	0.21	0.791	0.0062	NA	NA	NA	NA	NA	NA
miR-20a-5p	LGSOC	0.1306	0.8065	1.00 × 10^−4^	0.9578	NA	NA	NA	NA	NA
miR-20a-5p	SBT	0.8902	0.1896	0.0027	0.2796	0.2449	NA	NA	NA	NA
miR-20a-5p	BSC	0.7689	0.1523	0.0119	0.2496	0.0804	0.8187	NA	NA	NA
miR-20a-5p	Ovarian endometrioma	0.8646	0.2171	0.0362	0.2561	0.1971	0.8079	0.8801	NA	NA
miR-20a-5p	Deep infiltrating endometriosis	0.4696	0.0529	0.0021	0.1025	0.1072	0.8841	0.8428	0.9759	NA

* The table shows the values of the statistical significance of the differences between the compared groups using the two-tailed Wilcoxon–Mann–Whitney test.

## Data Availability

Not applicable.

## Supplementary Materials

The following supporting information can be downloaded at: https://www.mdpi.com/article/10.3390/life12122017/s1, Table S1: NGS data on read counts of miRNAs in tumor tissues.

## References

[1] Reid B.M., Permuth J.B., Sellers T.A. (2017). Epidemiology of ovarian cancer: A review. Cancer Biol. Med..

[2] Seidman J.D., Kurman R.J. (2003). Pathology of ovarian carcinoma. Hematol. Oncol. Clin. N. Am..

[3] Shih I.-M., Kurman R.J. (2004). Ovarian tumorigenesis: A proposed model based on morphological and molecular genetic analysis. Am. J. Pathol..

[4] Levanon K., Crum C., Drapkin R. (2008). New insights into the pathogenesis of serous ovarian cancer and its clinical impact. J. Clin. Oncol. Off. J. Am. Soc. Clin. Oncol..

[5] (2011). Integrated genomic analyses of ovarian carcinoma. Nature.

[6] Kanchi K.L., Johnson K.J., Lu C., McLellan M.D., Leiserson M.D.M., Wendl M.C., Zhang Q., Koboldt D.C., Xie M., Kandoth C. (2014). Integrated analysis of germline and somatic variants in ovarian cancer. Nat. Commun..

[7] Yang D., Sun Y., Hu L., Zheng H., Ji P., Pecot C.V., Zhao Y., Reynolds S., Cheng H., Rupaimoole R. (2013). Integrated analyses identify a master microRNA regulatory network for the mesenchymal subtype in serous ovarian cancer. Cancer Cell.

[8] Song X., Ji J., Gleason K.J., Yang F., Martignetti J.A., Chen L.S., Wang P. (2019). Insights into Impact of DNA Copy Number Alteration and Methylation on the Proteogenomic Landscape of Human Ovarian Cancer via a Multi-omics Integrative Analysis. Mol. Cell. Proteomics.

[9] Wrzeszczynski K.O., Varadan V., Byrnes J., Lum E., Kamalakaran S., Levine D.A., Dimitrova N., Zhang M.Q., Lucito R. (2011). Identification of tumor suppressors and oncogenes from genomic and epigenetic features in ovarian cancer. PLoS ONE.

[10] Garziera M., Cecchin E., Canzonieri V., Sorio R., Giorda G., Scalone S., De Mattia E., Roncato R., Gagno S., Poletto E. (2018). Identification of Novel Somatic TP53 Mutations in Patients with High-Grade Serous Ovarian Cancer (HGSOC) Using Next-Generation Sequencing (NGS). Int. J. Mol. Sci..

[11] Jones S., Wang T.-L., Kurman R.J., Nakayama K., Velculescu V.E., Vogelstein B., Kinzler K.W., Papadopoulos N., Shih I.-M. (2012). Low-grade serous carcinomas of the ovary contain very few point mutations. J. Pathol..

[12] Dey P., Nakayama K., Razia S., Ishikawa M., Ishibashi T., Yamashita H., Kanno K., Sato S., Kiyono T., Kyo S. (2022). Development of Low-Grade Serous Ovarian Carcinoma from Benign Ovarian Serous Cystadenoma Cells. Cancers.

[13] Sturgeon C.M., Duffy M.J., Stenman U.-H., Lilja H., Brünner N., Chan D.W., Babaian R., Bast R.C.J., Dowell B., Esteva F.J. (2008). National Academy of Clinical Biochemistry laboratory medicine practice guidelines for use of tumor markers in testicular, prostate, colorectal, breast, and ovarian cancers. Clin. Chem..

[14] Einhorn N., Sjövall K., Knapp R.C., Hall P., Scully R.E., Bast R.C.J., Zurawski V.R.J. (1992). Prospective evaluation of serum CA 125 levels for early detection of ovarian cancer. Obstet. Gynecol..

[15] Gerstein M. (2012). Genomics: ENCODE leads the way on big data. Nature.

[16] Sayed D., Abdellatif M. (2011). MicroRNAs in development and disease. Physiol. Rev..

[17] Calin G.A., Croce C.M. (2006). MicroRNA signatures in human cancers. Nat. Rev. Cancer.

[18] Wang F., Song X., Li X., Xin J., Wang S., Yang W., Wang J., Wu K., Chen X., Liang J. (2013). Noninvasive visualization of microRNA-16 in the chemoresistance of gastric cancer using a dual reporter gene imaging system. PLoS ONE.

[19] Petrick J.L., Pfeiffer R.M., Liao L.M., Abnet C.C., Wu X., Gammon M.D., Vaughan T.L., Cook M.B. (2021). Circulating MicroRNAs in Relation to Esophageal Adenocarcinoma Diagnosis and Survival. Dig. Dis. Sci..

[20] Gatto L., Franceschi E., Di Nunno V., Tosoni A., Lodi R., Brandes A.A. (2021). Liquid Biopsy in Glioblastoma Management: From Current Research to Future Perspectives. Oncologist.

[21] Van Laar R., King S., McCoy R., Saad M., Fereday S., Winship I., Uzzell C., Landgren A. (2021). Translation of a circulating miRNA signature of melanoma into a solid tissue assay to improve diagnostic accuracy and precision. Biomark. Med..

[22] Izumi D., Zhu Z., Chen Y., Toden S., Huo X., Kanda M., Ishimoto T., Gu D., Tan M., Kodera Y. (2021). Assessment of the Diagnostic Efficiency of a Liquid Biopsy Assay for Early Detection of Gastric Cancer. JAMA Netw. Open.

[23] Abe S., Matsuzaki J., Sudo K., Oda I., Katai H., Kato K., Takizawa S., Sakamoto H., Takeshita F., Niida S. (2021). A novel combination of serum microRNAs for the detection of early gastric cancer. Gastric Cancer.

[24] Ueta E., Tsutsumi K., Kato H., Matsushita H., Shiraha H., Fujii M., Matsumoto K., Horiguchi S., Okada H. (2021). Extracellular vesicle-shuttled miRNAs as a diagnostic and prognostic biomarker and their potential roles in gallbladder cancer patients. Sci. Rep..

[25] Dahiya N., Sherman-Baust C.A., Wang T.-L., Davidson B., Shih I.-M., Zhang Y., Wood W., Becker K.G., Morin P.J. (2008). MicroRNA expression and identification of putative miRNA targets in ovarian cancer. PLoS ONE.

[26] Iorio M.V., Visone R., Di Leva G., Donati V., Petrocca F., Casalini P., Taccioli C., Volinia S., Liu C.-G., Alder H. (2007). MicroRNA signatures in human ovarian cancer. Cancer Res..

[27] Zhang L., Volinia S., Bonome T., Calin G.A., Greshock J., Yang N., Liu C.-G., Giannakakis A., Alexiou P., Hasegawa K. (2008). Genomic and epigenetic alterations deregulate microRNA expression in human epithelial ovarian cancer. Proc. Natl. Acad. Sci. USA.

[28] Wyman S.K., Parkin R.K., Mitchell P.S., Fritz B.R., O’Briant K., Godwin A.K., Urban N., Drescher C.W., Knudsen B.S., Tewari M. (2009). Repertoire of microRNAs in epithelial ovarian cancer as determined by next generation sequencing of small RNA cDNA libraries. PLoS ONE.

[29] Prat J. (2015). Pathology of cancers of the female genital tract. Int. J. Gynaecol. Obstet..

[30] Perets R., Wyant G.A., Muto K.W., Bijron J.G., Poole B.B., Chin K.T., Chen J.Y.H., Ohman A.W., Stepule C.D., Kwak S. (2013). Transformation of the fallopian tube secretory epithelium leads to high-grade serous ovarian cancer in Brca;Tp53;Pten models. Cancer Cell.

[31] Poole E.M., Rice M.S., Crum C.P., Tworoger S.S. (2015). Salpingectomy as a potential ovarian cancer risk-reducing procedure. J. Natl. Cancer Inst..

[32] Koshiyama M., Matsumura N., Konishi I. (2014). Recent concepts of ovarian carcinogenesis: Type I and type II. BioMed Res. Int..

[33] Langmead B., Trapnell C., Pop M., Salzberg S.L. (2009). Ultrafast and memory-efficient alignment of short DNA sequences to the human genome. Genome Biol..

[34] Team R.C. (2021). A Language and Environment for Statistical Computing.

[35] Love M.I., Huber W., Anders S. (2014). Moderated estimation of fold change and dispersion for RNA-seq data with DESeq2. Genome Biol..

[36] Daltoé R.D., Madeira K.P., de Carvalho A.A., de Rezende L.C.D., Silva I.V., Rangel L.B.A. (2014). Evaluation of the progesterone receptor status in breast cancer using three different antibodies: A comparison by Allred score system. Int. J. Clin. Exp. Pathol..

[37] R Team RStudio: Integrated Development for R. RStudio. http://www.rstudio.com/.

[38] Mutch D.G., Prat J. (2014). 2014 FIGO staging for ovarian, fallopian tube and peritoneal cancer. Gynecol. Oncol..

[39] Carcangiu Kurman R.J., Carcangiu M.L., Herrington C., Simon M.L. (2014). WHO Classification of Tumours of Female Reproductive Organs. https://publications.iarc.fr/Book-And-Report-Series/Who-Classification-Of-Tumours/WHO-Classification-Of-Tumours-Of-Female-Reproductive-Organs-2014.

[40] Yang Y.-M., Yang W.-X. (2017). Epithelial-to-mesenchymal transition in the development of endometriosis. Oncotarget.

[41] Nick A.M., Coleman R.L., Ramirez P.T., Sood A.K. (2015). A framework for a personalized surgical approach to ovarian cancer. Nat. Rev. Clin. Oncol..

[42] Schorge J.O., McCann C., Del Carmen M.G. (2010). Surgical debulking of ovarian cancer: What difference does it make?. Rev. Obstet. Gynecol..

[43] Borley J., Wilhelm-Benartzi C., Brown R., Ghaem-Maghami S. (2012). Does tumour biology determine surgical success in the treatment of epithelial ovarian cancer? A systematic literature review. Br. J. Cancer.

[44] van Driel W.J., Koole S.N., Sikorska K., Schagen van Leeuwen J.H., Schreuder H.W.R., Hermans R.H.M., de Hingh I.H.J.T., van der Velden J., Arts H.J., Massuger L.F.A.G. (2018). Hyperthermic Intraperitoneal Chemotherapy in Ovarian Cancer. N. Engl. J. Med..

[45] Caratozzolo M.F., Micale L., Turturo M.G., Cornacchia S., Fusco C., Marzano F., Augello B., D’Erchia A.M., Guerrini L., Pesole G. (2012). TRIM8 modulates p53 activity to dictate cell cycle arrest. Cell Cycle.

[46] Vogelstein B., Lane D., Levine A.J. (2000). Surfing the p53 network. Nature.

[47] Lane D., Levine A. (2010). p53 Research: The past thirty years and the next thirty years. Cold Spring Harb. Perspect. Biol..

[48] Riley T., Sontag E., Chen P., Levine A. (2008). Transcriptional control of human p53-regulated genes. Nat. Rev. Mol. Cell Biol..

[49] Mastropasqua F., Marzano F., Valletti A., Aiello I., Di Tullio G., Morgano A., Liuni S., Ranieri E., Guerrini L., Gasparre G. (2017). TRIM8 restores p53 tumour suppressor function by blunting N-MYC activity in chemo-resistant tumours. Mol. Cancer.

[50] Wang Z., Ji F. (2018). Downregulation of microRNA-17-5p inhibits drug resistance of gastric cancer cells partially through targeting p21. Oncol. Lett..

[51] Mauro L.J., Seibel M.I., Diep C.H., Spartz A., Perez Kerkvliet C., Singhal H., Swisher E.M., Schwartz L.E., Drapkin R., Saini S. (2021). Progesterone Receptors Promote Quiescence and Ovarian Cancer Cell Phenotypes via DREAM in p53-Mutant Fallopian Tube Models. J. Clin. Endocrinol. Metab..

[52] Wang X., Wei P., Yang L., Liu F., Tong X., Yang X., Su L. (2022). MicroRNA-20a-5p regulates the epithelial-mesenchymal transition of human hepatocellular carcinoma by targeting *RUNX3*. Chin. Med. J..

[53] Tanaka S., Shiraha H., Nakanishi Y., Nishina S.-I., Matsubara M., Horiguchi S., Takaoka N., Iwamuro M., Kataoka J., Kuwaki K. (2012). Runt-related transcription factor 3 reverses epithelial-mesenchymal transition in hepatocellular carcinoma. Int. J. Cancer.

[54] Xiao Z., Tian Y., Jia Y., Shen Q., Jiang W., Chen G., Shang B., Shi M., Wang Z., Zhao X. (2020). RUNX3 inhibits the invasion and migration of esophageal squamous cell carcinoma by reversing the epithelial-mesenchymal transition through TGF-β/Smad signaling. Oncol. Rep..

[55] Cheng D., Zhao S., Tang H., Zhang D., Sun H., Yu F., Jiang W., Yue B., Wang J., Zhang M. (2016). MicroRNA-20a-5p promotes colorectal cancer invasion and metastasis by downregulating Smad4. Oncotarget.

[56] Zhang B., Halder S.K., Kashikar N.D., Cho Y.-J., Datta A., Gorden D.L., Datta P.K. (2010). Antimetastatic role of Smad4 signaling in colorectal cancer. Gastroenterology.

[57] Park J.W., Jang S.H., Park D.M., Lim N.J., Deng C., Kim D.Y., Green J.E., Kim H.K. (2014). Cooperativity of E-cadherin and Smad4 loss to promote diffuse-type gastric adenocarcinoma and metastasis. Mol. Cancer Res..

[58] Li J., Yang S., Yan W., Yang J., Qin Y.-J., Lin X.-L., Xie R.-Y., Wang S.-C., Jin W., Gao F. (2015). MicroRNA-19 triggers epithelial-mesenchymal transition of lung cancer cells accompanied by growth inhibition. Lab. Investig..

[59] Bao C., Liu T., Qian L., Xiao C., Zhou X., Ai H., Wang J., Fan W., Pan J. (2021). Shikonin inhibits migration and invasion of triple-negative breast cancer cells by suppressing epithelial-mesenchymal transition via miR-17-5p/PTEN/Akt pathway. J. Cancer.

[60] Khanbabaei H., Ebrahimi S., García-Rodríguez J.L., Ghasemi Z., Pourghadamyari H., Mohammadi M., Kristensen L.S. (2022). Non-coding RNAs and epithelial mesenchymal transition in cancer: Molecular mechanisms and clinical implications. J. Exp. Clin. Cancer Res..

[61] Thiery J.P., Sleeman J.P. (2006). Complex networks orchestrate epithelial-mesenchymal transitions. Nat. Rev. Mol. Cell Biol..

[62] Evdokimova V., Tognon C., Ng T., Sorensen P.H.B. (2009). Reduced proliferation and enhanced migration: Two sides of the same coin? Molecular mechanisms of metastatic progression by YB-1. Cell Cycle.

[63] Polyak K., Weinberg R.A. (2009). Transitions between epithelial and mesenchymal states: Acquisition of malignant and stem cell traits. Nat. Rev. Cancer.

[64] Zhou Z., Wang X., Ren X., Zhou L., Wang N., Kang H. (2021). Disease Burden and Attributable Risk Factors of Ovarian Cancer From 1990 to 2017: Findings From the Global Burden of Disease Study 2017. Front. Public Health.

[65] Fabbro M., Colombo P.-E., Leaha C.M., Rouanet P., Carrère S., Quenet F., Gutowski M., Mourregot A., D’Hondt V., Coupier I. (2020). Conditional Probability of Survival and Prognostic Factors in Long-Term Survivors of High-Grade Serous Ovarian Cancer. Cancers.

[66] Bowtell D.D., Böhm S., Ahmed A.A., Aspuria P.-J., Bast R.C.J., Beral V., Berek J.S., Birrer M.J., Blagden S., Bookman M.A. (2015). Rethinking ovarian cancer II: Reducing mortality from high-grade serous ovarian cancer. Nat. Rev. Cancer.

[67] Kurman R.J. (2014). WHO classification of tumours of female reproductive organs. World Health Organization Classification of Tumours.

[68] Kuhn E., Kurman R.J., Vang R., Sehdev A.S., Han G., Soslow R., Wang T.-L., Shih I.-M. (2012). TP53 mutations in serous tubal intraepithelial carcinoma and concurrent pelvic high-grade serous carcinoma—Evidence supporting the clonal relationship of the two lesions. J. Pathol..

[69] Chen F., Gaitskell K., Garcia M.J., Albukhari A., Tsaltas J., Ahmed A.A. (2017). Serous tubal intraepithelial carcinomas associated with high-grade serous ovarian carcinomas: A systematic review. BJOG Int. J. Obstet. Gynaecol..

[70] Cheng E.J., Kurman R.J., Wang M., Oldt R., Wang B.G., Berman D.M., Shih I.-M. (2004). Molecular genetic analysis of ovarian serous cystadenomas. Lab. Investig..

[71] Kurman R.J., Vang R., Junge J., Hannibal C.G., Kjaer S.K., Shih I.-M. (2011). Papillary tubal hyperplasia: The putative precursor of ovarian atypical proliferative (borderline) serous tumors, noninvasive implants, and endosalpingiosis. Am. J. Surg. Pathol..

[72] Vang R., Shih I.-M., Kurman R.J. (2013). Fallopian tube precursors of ovarian low- and high-grade serous neoplasms. Histopathology.

[73] Wolsky R.J., Price M.A., Zaloudek C.J., Rabban J.T. (2018). Mucosal Proliferations in Completely Examined Fallopian Tubes Accompanying Ovarian Low-grade Serous Tumors: Neoplastic Precursor Lesions or Normal Variants of Benign Mucosa?. Int. J. Gynecol. Pathol..

[74] Liu Z., Beach J.A., Agadjanian H., Jia D., Aspuria P.-J., Karlan B.Y., Orsulic S. (2015). Suboptimal cytoreduction in ovarian carcinoma is associated with molecular pathways characteristic of increased stromal activation. Gynecol. Oncol..

[75] Riester M., Wei W., Waldron L., Culhane A.C., Trippa L., Oliva E., Kim S.-H., Michor F., Huttenhower C., Parmigiani G. (2014). Risk prediction for late-stage ovarian cancer by meta-analysis of 1525 patient samples. J. Natl. Cancer Inst..

[76] Tucker S.L., Gharpure K., Herbrich S.M., Unruh A.K., Nick A.M., Crane E.K., Coleman R.L., Guenthoer J., Dalton H.J., Wu S.Y. (2014). Molecular biomarkers of residual disease after surgical debulking of high-grade serous ovarian cancer. Clin. Cancer Res..

[77] Ryner L., Guan Y., Firestein R., Xiao Y., Choi Y., Rabe C., Lu S., Fuentes E., Huw L.-Y., Lackner M.R. (2015). Upregulation of Periostin and Reactive Stroma Is Associated with Primary Chemoresistance and Predicts Clinical Outcomes in Epithelial Ovarian Cancer. Clin. Cancer Res..

[78] Wittmann J., Jäck H.-M. (2010). Serum microRNAs as powerful cancer biomarkers. Biochim. Biophys. Acta.

[79] Shih K.K., Qin L.-X., Tanner E.J., Zhou Q., Bisogna M., Dao F., Olvera N., Viale A., Barakat R.R., Levine D.A. (2011). A microRNA survival signature (MiSS) for advanced ovarian cancer. Gynecol. Oncol..

[80] Tang Z., Ow G.S., Thiery J.P., Ivshina A.V., Kuznetsov V.A. (2014). Meta-analysis of transcriptome reveals let-7b as an unfavorable prognostic biomarker and predicts molecular and clinical subclasses in high-grade serous ovarian carcinoma. Int. J. Cancer.

[81] Bhaskaran M., Mohan M. (2014). MicroRNAs: History, biogenesis, and their evolving role in animal development and disease. Vet. Pathol..

[82] Bartel D.P. (2004). MicroRNAs: Genomics, biogenesis, mechanism, and function. Cell.

[83] Kuznetsov V.A., Tang Z., Ivshina A. (2017). V Identification of common oncogenic and early developmental pathways in the ovarian carcinomas controlling by distinct prognostically significant microRNA subsets. BMC Genom..

[84] Ahmed N., Abubaker K., Findlay J., Quinn M. (2010). Epithelial mesenchymal transition and cancer stem cell-like phenotypes facilitate chemoresistance in recurrent ovarian cancer. Curr. Cancer Drug Targets.

[85] Marchini S., Fruscio R., Clivio L., Beltrame L., Porcu L., Fuso Nerini I., Cavalieri D., Chiorino G., Cattoretti G., Mangioni C. (2013). Resistance to platinum-based chemotherapy is associated with epithelial to mesenchymal transition in epithelial ovarian cancer. Eur. J. Cancer.

[86] Alvero A.B., Chen R., Fu H.-H., Montagna M., Schwartz P.E., Rutherford T., Silasi D.-A., Steffensen K.D., Waldstrom M., Visintin I. (2009). Molecular phenotyping of human ovarian cancer stem cells unravels the mechanisms for repair and chemoresistance. Cell Cycle.

[87] Yin G., Chen R., Alvero A.B., Fu H.-H., Holmberg J., Glackin C., Rutherford T., Mor G. (2010). TWISTing stemness, inflammation and proliferation of epithelial ovarian cancer cells through MIR199A2/214. Oncogene.

[88] Xi X.-P., Zhuang J., Teng M.-J., Xia L.-J., Yang M.-Y., Liu Q.-G., Chen J.-B. (2016). MicroRNA-17 induces epithelial-mesenchymal transition consistent with the cancer stem cell phenotype by regulating CYP7B1 expression in colon cancer. Int. J. Mol. Med..

[89] Fang Y., Xu C., Fu Y. (2015). MicroRNA-17-5p induces drug resistance and invasion of ovarian carcinoma cells by targeting PTEN signaling. J. Biol. Res..

[90] Nam E.J., Yoon H., Kim S.W., Kim H., Kim Y.T., Kim J.H., Kim J.W., Kim S. (2008). MicroRNA expression profiles in serous ovarian carcinoma. Clin. Cancer Res..

[91] Saral M.A., Tuncer S.B., Odemis D.A., Erdogan O.S., Erciyas S.K., Saip P., Ozel S., Yazici H. (2022). New biomarkers in peripheral blood of patients with ovarian cancer: High expression levels of miR-16-5p, miR-17-5p, and miR-638. Arch. Gynecol. Obstet..

[92] Resnick K.E., Alder H., Hagan J.P., Richardson D.L., Croce C.M., Cohn D.E. (2009). The detection of differentially expressed microRNAs from the serum of ovarian cancer patients using a novel real-time PCR platform. Gynecol. Oncol..

[93] Shah J.S., Gard G.B., Yang J., Maidens J., Valmadre S., Soon P.S., Marsh D.J. (2018). Combining serum microRNA and CA-125 as prognostic indicators of preoperative surgical outcome in women with high-grade serous ovarian cancer. Gynecol. Oncol..

[94] Comamala M., Pinard M., Thériault C., Matte I., Albert A., Boivin M., Beaudin J., Piché A., Rancourt C. (2011). Downregulation of cell surface CA125/MUC16 induces epithelial-to-mesenchymal transition and restores EGFR signalling in NIH:OVCAR3 ovarian carcinoma cells. Br. J. Cancer.

[95] Zhang Q., Wang Q., Sun W., Gao F., Liu L., Cheng L., Li Z. (2018). Change of Circulating and Tissue-Based miR-20a in Human Cancers and Associated Prognostic Implication: A Systematic Review and Meta-Analysis. BioMed Res. Int..

[96] Du Y., Zhu M., Zhou X., Huang Z., Zhu J., Xu J., Cheng G., Shu Y., Liu P., Zhu W. (2016). miR-20a enhances cisplatin resistance of human gastric cancer cell line by targeting NFKBIB. Tumour Biol..

[97] Wang Z., Zhang J., Zhang Z., Jiang Y., Li M., Li Q., Bai L., Yao D., Wang M., Wang X. (2018). Prognostic value of miR-17-5 p in gastrointestinal cancers: A systematic review and meta-analysis. OncoTargets. Ther..

[98] Mogilyansky E., Rigoutsos I. (2013). The miR-17/92 cluster: A comprehensive update on its genomics, genetics, functions and increasingly important and numerous roles in health and disease. Cell Death Differ..

[99] Xu X., Zhu S., Tao Z., Ye S. (2018). High circulating miR-18a, miR-20a, and miR-92a expression correlates with poor prognosis in patients with non-small cell lung cancer. Cancer Med..

[100] Wang M., Gu H., Wang S., Qian H., Zhu W., Zhang L., Zhao C., Tao Y., Xu W. (2012). Circulating miR-17-5p and miR-20a: Molecular markers for gastric cancer. Mol. Med. Rep..

[101] Palmigiano A., Santaniello F., Cerutti A., Penkov D., Purushothaman D., Makhija E., Luzi L., di Fagagna F.D., Pelicci P.G., Shivashankar V. (2018). PREP1 tumor suppressor protects the late-replicating DNA by controlling its replication timing and symmetry. Sci. Rep..

[102] Longobardi E., Iotti G., Di Rosa P., Mejetta S., Bianchi F., Fernandez-Diaz L.C., Micali N., Nuciforo P., Lenti E., Ponzoni M. (2010). Prep1 (pKnox1)-deficiency leads to spontaneous tumor development in mice and accelerates EmuMyc lymphomagenesis: A tumor suppressor role for Prep1. Mol. Oncol..

[103] Briu L.-M., Maric C., Cadoret J.-C. (2021). Replication Stress, Genomic Instability, and Replication Timing: A Complex Relationship. Int. J. Mol. Sci..

[104] Wu N.-Y., Huang H.-S., Chao T.H., Chou H.M., Fang C., Qin C.-Z., Lin C.-Y., Chu T.-Y., Zhou H.H. (2017). Progesterone Prevents High-Grade Serous Ovarian Cancer by Inducing Necroptosis of p53-Defective Fallopian Tube Epithelial Cells. Cell Rep..

[105] Tone A.A., Virtanen C., Shaw P.A., Brown T.J. (2011). Decreased progesterone receptor isoform expression in luteal phase fallopian tube epithelium and high-grade serous carcinoma. Endocr. Relat. Cancer.

[106] Li L., Gu H., Chen L., Zhu P., Zhao L., Wang Y., Zhao X., Zhang X., Zhang Y., Shu P. (2019). Integrative Network Analysis Reveals a MicroRNA-Based Signature for Prognosis Prediction of Epithelial Ovarian Cancer. BioMed Res. Int..

[107] Zhao L., Wang W., Xu L., Yi T., Zhao X., Wei Y., Vermeulen L., Goel A., Zhou S., Wang X. (2019). Integrative network biology analysis identifies miR-508-3p as the determinant for the mesenchymal identity and a strong prognostic biomarker of ovarian cancer. Oncogene.

[108] Yoshida K., Yokoi A., Matsuzaki J., Kato T., Ochiya T., Kajiyama H., Yamamoto Y. (2021). Extracellular microRNA profiling for prognostic prediction in patients with high-grade serous ovarian carcinoma. Cancer Sci..

[109] Lopacinska-Jørgensen J., Oliveira D.V.N.P., Wayne Novotny G., Høgdall C.K., Høgdall E. (2021). V Integrated microRNA and mRNA signatures associated with overall survival in epithelial ovarian cancer. PLoS ONE.

